# Increased left fusiform surface area: a structural predisposition to ticker-tape synesthesia

**DOI:** 10.1093/braincomms/fcag408

**Published:** 2026-10-27

**Authors:** Romain Lahbari, Romain Delsanti, Romain Valabrègue, Yusaku Takamura, Arnaud Cachia, Fabien Hauw, Laurent Cohen

**Affiliations:** Inserm U 1127, CNRS UMR 7225, Sorbonne Universités, Institut du Cerveau, ICM, Paris 75013, France; Inserm U 1127, CNRS UMR 7225, Sorbonne Universités, Institut du Cerveau, ICM, Paris 75013, France; Inserm U 1127, CNRS UMR 7225, Sorbonne Universités, Institut du Cerveau, ICM, Paris 75013, France; Inserm U 1127, CNRS UMR 7225, Sorbonne Universités, Institut du Cerveau, ICM, Paris 75013, France; Neurorehabilitation Research Center, Kio University, Nara 635-0832, Japan; Université Paris Cité, Laboratoire de Psychologie du Développement et de L'Education, UMR CNRS 8240, La Sorbonne, Paris 75005, France; Université Paris Cité, Imaging Biomarkers for Brain Development and Disorders, UMR INSERM 1266, GHU Paris Psychiatrie & Neurosciences, Paris 75014, France; Inserm U 1127, CNRS UMR 7225, Sorbonne Universités, Institut du Cerveau, ICM, Paris 75013, France; Inserm U 1127, CNRS UMR 7225, Sorbonne Universités, Institut du Cerveau, ICM, Paris 75013, France; AP-HP, Hôpital de La Pitié Salpêtrière, Institut de Neurologie, Paris 75013, France

**Keywords:** synesthesia, dyslexia, reading, language, cortical morphometry

## Abstract

Ticker-tape synesthesia (TTS) is a developmental variant in which individuals perceive in their mind’s eye the written form of heard speech vividly and automatically. Previous work documented functional over-activation of the reading network during speech perception, increased functional connectivity between the Visual Word Form Area (VWFA) and left temporoparietal regions and increased anatomical connectivity through the arcuate fasciculus in synesthetes. Here, we investigated cortical morphology in 17 synesthetes and 17 matched controls. Synesthetes showed significantly increased cortical surface area in the left fusiform gyrus, centred on the mid-fusiform gyrus and extending towards the VWFA. No group difference was found in cortical thickness or curvature. This establishes cortical morphology as a further line of evidence implicating the left ventral occipitotemporal cortex in TTS. Three arguments suggest that this area increase reflects an innate predisposition rather than a consequence of the synesthetic experience: cortical surface area, unlike thickness, is more closely related to early neurodevelopmental processes; reduced area in the same region characterizes developmental dyslexia and the surface increase extends beyond the reading network. We propose that an innate expansion of fusiform cortex provides a richer substrate for orthographic representations, excessively activated by top-down phonological input, giving rise to the involuntary visualization of speech.

## Teaser

Lahbari *et al*. report that individuals with ticker-tape synesthesia, who involuntarily visualize the written form of heard speech, have significantly greater cortical surface area in the left fusiform gyrus than matched controls. This structural difference, likely of prenatal origin, may constitute an innate predisposition for orthographic visualization of speech.

## Graphical Abstract

**Graphical Abstract:**
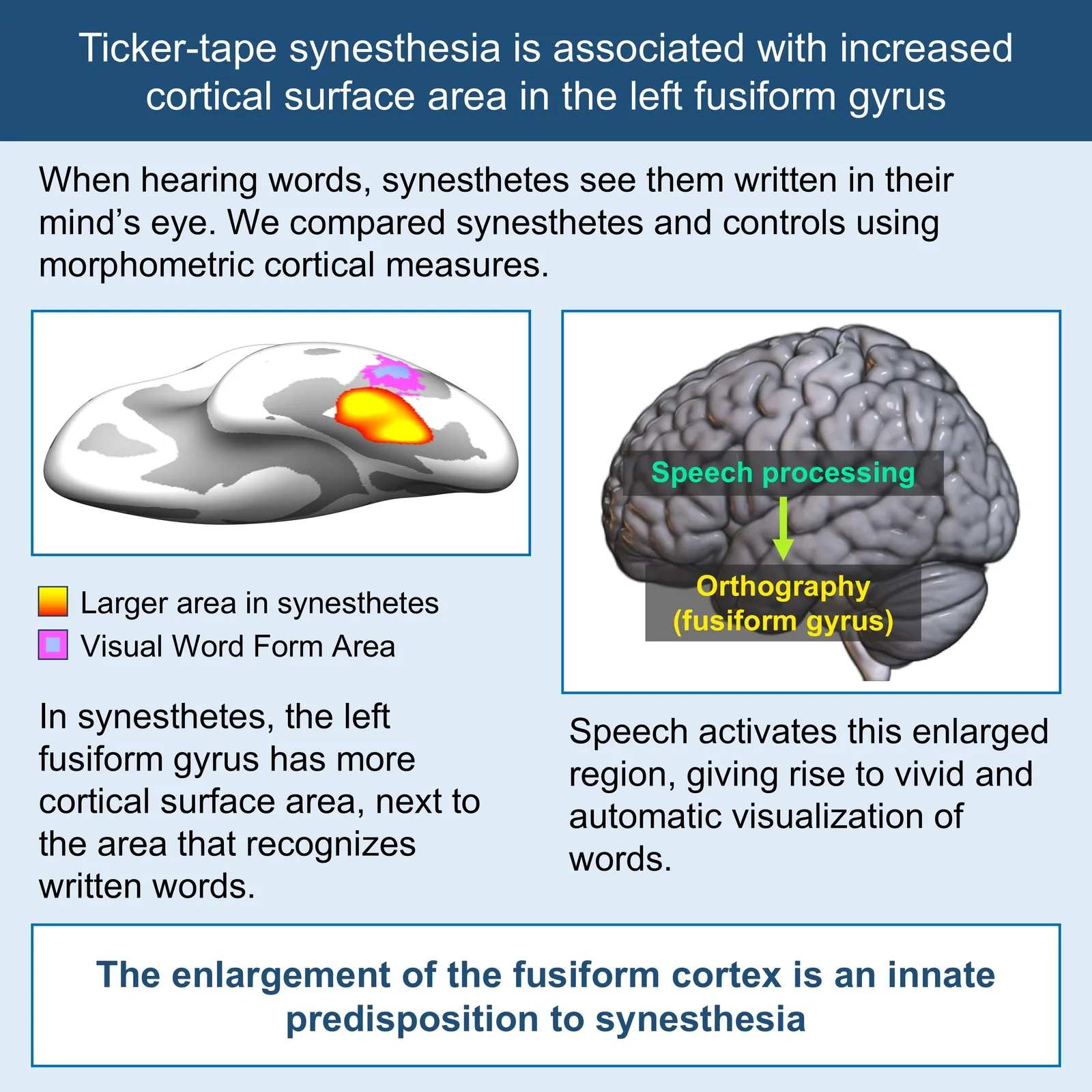

## Introduction

Ticker-tape synesthesia is a developmental form of synesthesia in which individuals vividly and automatically perceive, in their mind’s eye, the written form of heard speech, as if subtitles were scrolling in their mind’s eye. It was first described by Francis Galton in the late 19th century^1^ and then fell out of interest for a century.^2^ In the vast majority of synesthetes, the orthographic percept is experienced in the mind’s eye rather than projected into external space, while a few synesthetes report projector-like qualities.^3^ This phenomenon engages the core reading network, and in particular two regions that are critical for the crossmodal integration of sounds and letters: the posterior superior temporal cortex and supramarginal gyrus (pSTG/SMG), where speech sounds interface with other modalities, and the Visual Word Form Area (VWFA), a reliably localized region of the left ventral occipitotemporal cortex (VOTC) that supports the visual recognition of letter strings.^4,5^

Functional MRI evidence indicates that, during speech perception, ticker-tape synesthetes show greater activation than controls in both the pSTG/SMG and the VWFA, and that these two regions are functionally over-connected with each other, consistent with the hypothesis that TTS results from an increased top-down phonological influence on orthographic representations.^5^ In a recent study, we reported that TTS is also associated with increased anatomical connectivity through the posterior segment of the arcuate fasciculus, the white matter pathway that links the pSTG/SMG to the VWFA.^6^ Beyond the core reading network, including the VWFA and left perisylvian language regions, there is also increased functional connectivity at rest between left prefrontal areas and the bilateral occipital cortex, a possible correlate of a predisposition to all synesthesias with a visual component.^5^ In simple terms, the hypothesis is that heard speech automatically recruits brain systems normally used for seeing written words, causing speech to be experienced with an internally generated visual orthographic form.

TTS belongs to the broad family of synesthesias, in which stimulation of one sensory or cognitive domain automatically triggers a concurrent experience in another domain.^7,8^ As in TTS, increased connectivity has been put forward repeatedly as a core component of grapheme-colour^9,10^ and other synesthesias.^11^ What distinguishes TTS from other synesthetic phenomena is that both the trigger and the concurrent are high-level orthographic and phonological language representations rather than an elementary sensory quality such as a colour or a shape, a feature that anchors it within the cognitive neuroscience of language and reading.

The convergent findings reviewed before point to the left VOTC as a critical node in the network of areas involved in TTS. However, it remains unknown whether TTS is also associated with differences in cortical morphology, which could constitute a further structural substrate predisposing certain individuals to develop this form of synesthesia. The existence of differences in cortical morphology is plausible considering that such differences exist in the two most studied types of synesthesia. Thus, persons with absolute pitch have increased cortical volume^12^ and thickness^13^ in the superior temporal gyrus. In grapheme-colour synesthesia, which shares with TTS atypical orthographic mental imagery, there is increased cortical thickness, volume and surface area in the fusiform gyri and adjacent regions.^14,15^

Here, we analysed cortical surface area, thickness and curvature in the same participants who were studied by Delsanti *et al*.^6^ Cortical surface area reflects the number of cortical columns and is primarily determined by prenatal proliferation of progenitor cells; cortical thickness reflects postnatal processes including synaptic pruning and myelination and cortical curvature indexes the folding pattern of the cortex, itself partly determined by mechanical and connectivity-related constraints during development.^16,17^ These three measures are genetically and developmentally dissociable, which motivated their separate examination in a case in which neural predispositions and learning (here reading acquisition) interact.

Based on the functional and connectional evidence reviewed above, we predicted that synesthetes would show morphological differences in two candidate regions, the left VOTC and the left pSTG/SMG.

## Materials and methods

### Participants

We studied 17 ticker-tape synesthetes and 17 matched controls, the same participants as in Delsanti *et al*. (2025).^6^ The T1-weighted anatomical images analysed here were acquired during the same scanning sessions as the data reported in Delsanti *et al*. (2025),^6^ but the morphometric analysis is entirely new. No overlap exists between the analyses reported here and those previously published. All participants were native French speakers, right-handed according to the Edinburgh Inventory and had no history of neurological or psychiatric disorders. Participants reported no history of dyslexia or reading disorder. Participants were matched one-to-one between groups in gender (14 females, 3 males per group), in age (synesthetes: mean 39.7 years, SD 13.9; controls: mean 38.6 years, SD 14.4; Student’s *t*-test with 32 df: *t* = 0.094, *P* = 0.83). All participants had higher education, and controls had approximately the same academic level as the corresponding synesthetes. Brain size did not differ between groups: total cortical surface area (*t* = 0.86, *P* = 0.398), supratentorial volume (*t* = 1.28, *P* = 0.209), cortical grey matter volume (Student’s *t*-test: *t* = 0.93, *P* = 0.360). A majority of synesthetes had other associated types of synesthesias, as previously reported in a partially overlapping group.^3^ The research was approved by the institutional review board of the INSERM (protocol C13-41), and all participants provided informed written consent in accordance with the Declaration of Helsinki.

### Image acquisition

All images were acquired on a Siemens 3T MAGNETOM Prisma scanner. T1-weighted anatomical images (TR/TE/flip angle = 2400 ms/2.23 ms/9°, voxel size = 0.7 × 0.7 × 0.7 mm) were acquired with a 64-channel receive-only head coil.

### Cortical surface reconstruction

Cortical surface reconstructions were generated using the FreeSurfer pipeline (version 7 Release 7.4.1) (https://surfer.nmr.mgh.harvard.edu/). We supplemented the segmentation of the brain by FreeSurfer with the AI segmentation model SIAM (github.com/romainVala/SIAM). SIAM generated an individual grey-matter mask segmenting the brain from the dura mater, vessels and skull with high accuracy. This mask was intersected with the grey-matter mask created by FreeSurfer at an early stage of the recon-all pipeline, removing voxels mistakenly labelled by FreeSurfer’s default segmentation. The use of SIAM compensated for the absence of T2 images, which may improve the reconstruction of the pial surface. The cleaned-up grey-matter mask was then input to the following stages of recon-all. Structural images, as well as the displays of the individual processing steps provided by FreeSurfer, were visually inspected for artefacts and segmentation quality. No participant was excluded on quality grounds. For each participant, cortical area, thickness and curvature were estimated at each vertex and resampled onto the fsaverage template (163 842 vertices per hemisphere). Surface maps were spatially smoothed using a Gaussian kernel with a full-width at half-maximum of 20 mm. The results were robust to the choice of smoothing kernel (see Supplementary Result S1, Supplementary Table S1, Supplementary Fig. S1).

### Statistical analysis

Vertex-wise statistical analyses were performed using a general linear model (GLM), with the BrainStat toolbox,^18^ implemented in MATLAB (MathWorks, Natick, MA). The design matrix included an intercept, a group factor, age (mean-centred), gender and two interaction terms (group × age, group × gender). Note that the group × gender interaction was included in the model to account for potential differential effects of gender across groups. Given the small number of male participants, this interaction term was severely underpowered, and its null result should not be interpreted as evidence of absence.

For each contrast of interest, t-statistics (df = 28) were computed at every vertex. Cluster-level inference was performed using random field theory (RFT^19^). Supra-threshold clusters were first defined at a cluster-forming threshold of *P* < 0.001 (uncorrected). Cluster-level *P*-values were then computed, yielding family-wise corrected significance at *P* < 0.05. For each significant cluster, we report the anatomical location, the RFT-corrected cluster-wise *P*-value, the peak *Z*-value, the cluster size (in vertices) and the centroid coordinates in MNI space (derived from the fsaverage pial surface).

### Definition of regions of interest

#### Fusiform ROIs

We performed a region-of-interest (ROI) analysis within the four cytoarchitectonic subdivisions (FG1–FG4) of the fusiform gyrus,^20,21^ projected on the pial surface. For each participant, the mean cortical area was computed within each subdivision, and group differences were assessed using a GLM with the same factors and interactions as in the whole-brain analysis.

#### Functional definition of the VWFA

The VWFA, as displayed in Fig. 1A and B, was defined in two congruent manners, as reported in Hauw *et al*. (2024)^5^: First from the contrast of words > other images (faces, houses and tools) during a simple viewing task; and second from the contrast of speech perception in synesthetes > controls.

**Figure 1:**
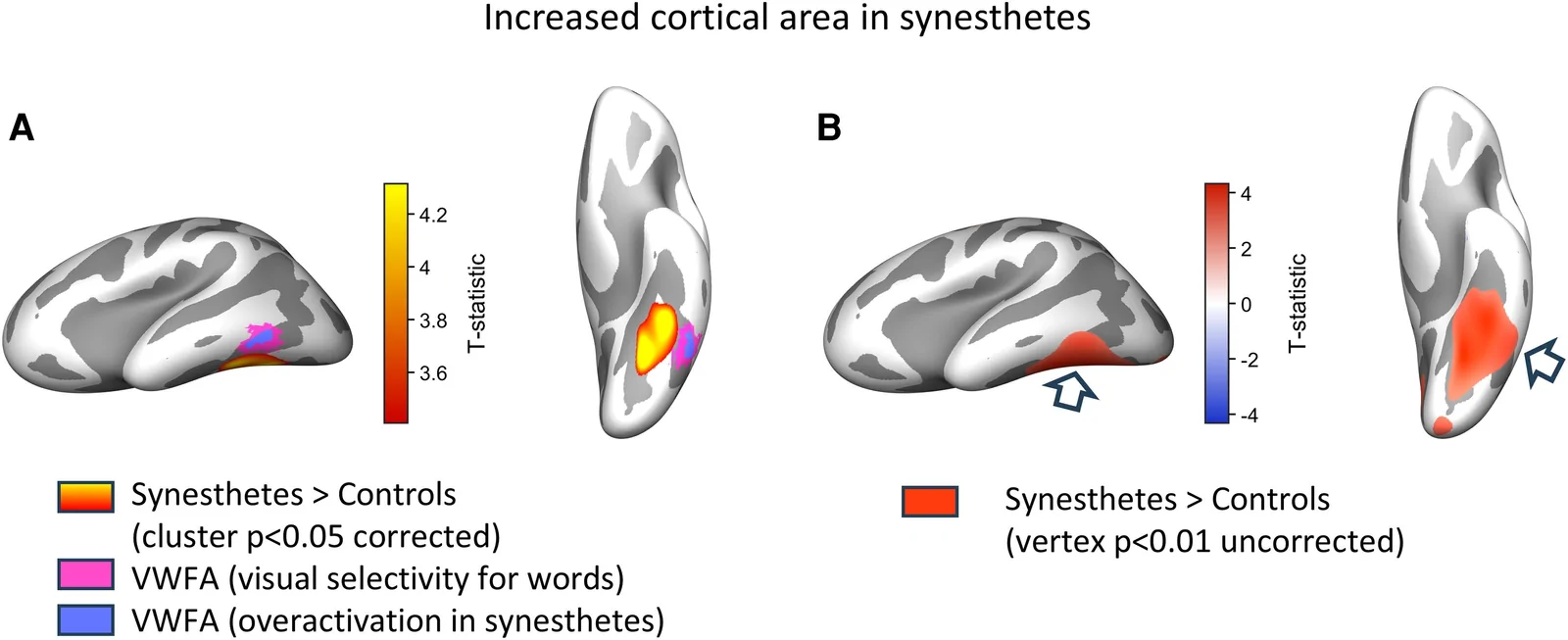
**Group differences in cortical surface area between synesthetes and controls (*N* = 17 per group).** Statistical analysis: vertex-wise general linear model including group, age, gender, group × age and group × gender, with a two-sample t-contrast comparing synesthetes and controls (df = 28). Cluster-level inference used random field theory (RFT). (**A**) Increased cortical surface area in the left fusiform gyrus of synesthetes relative to controls (hot; cluster-forming threshold *P* < 0.001, cluster-level threshold RFT-corrected *P* < 0.05; peak *Z* = 3.88, cluster-level corrected *P* = 0.0034), mesial to the Visual Word Form Area (VWFA) as defined by its selectivity for words (magenta) and by its over-activation during speech perception in synesthetes (blue). The colour bar indicates *t* values. (**B**) At a lower vertex-level threshold of *P* < 0.01 (red), this cluster overlaps with the VWFA (arrows).

#### Functional definition of the pSTG/SMG

The pSTG/SMG ROI was defined as the corresponding cluster identified in Hauw *et al*. (2024)^5^ from the contrast of speech perception in synesthetes > controls.

## Results

In 17 ticker-tape synesthetes and 17 matched controls, we studied cortical surface area, thickness and curvature. The analysis was conducted across the entire cortical surface, and significant effects were identified without a priori restriction to the regions involved in reading. We applied a vertex-wise cluster-forming threshold of *P* < 0.001, followed by cluster-wise correction for multiple comparisons across the whole cortical surface (corrected *P* < 0.05).

### Effect of synesthesia

Whole brain analysis revealed that cortical surface area was significantly larger in synesthetes than in controls in the left fusiform gyrus, centred on the mid-fusiform gyrus (Figs 1A and  2A; Table 1). This cluster was just mesial to the VWFA, as defined both by its increased activation in synesthetes relative tocontrols during speech perception, and by its activation by printed words relative to other images.^5^ To better appreciate the spatial relationship of the significant cluster with the VWFA, we lowered the vertex-wise threshold to *P* < 0.01 (uncorrected): the cluster of increased area then extended laterally into the occipitotemporal sulcus (OTS) to engulf the VWFA (Fig. 1B). This was done purely for descriptive and anatomical interpretation purposes, not to claim statistical significance at this threshold.

**Figure 2:**
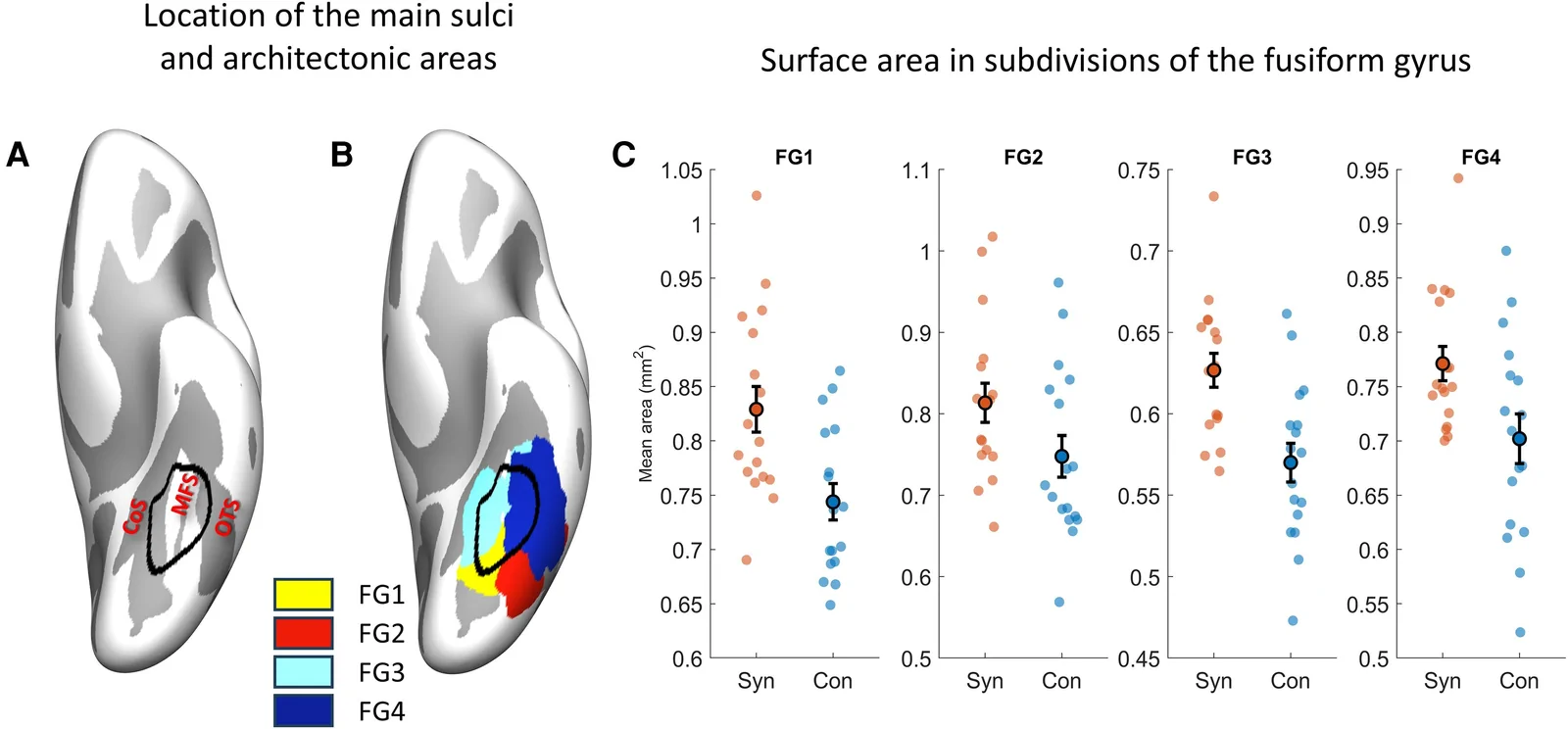
**Relations with anatomical structures.** (**A**) Location of the cluster of increased surface area in synesthetes (black outline, from the whole-brain vertex-wise GLM t-contrast shown in Fig. 1A; *N* = 17 per group) relative to the occipito-temporal sulcus (OTS), the mid-fusiform sulcus (MFS) and the collateral sulcus (CoS). (**B**) Location of the same cluster (black outline; *N* = 17 per group) relative to the four cytoarchitectonic subdivisions of the fusiform gyrus, FG1–FG4. (**C**) Individual average surface area in each subdivision of the fusiform gyrus for synesthetes (orange) and controls (blue). Each dot represents one participant (*N* = 17 per group). Error bars represent the SEM. Statistical analysis: repeated-measures ANOVA with Group as between-subject factor and ROI as within-subject factor, including age and gender as covariates. The Group × ROI interaction was not significant (*F*(3,84) = 0.81, *P* = 0.45).

**Table 1:** Whole-brain vertex-wise analysis of cortical surface area

	Cluster corrected *P* value	Effect size	Peak voxel *Z* value	Cluster size # vertices	Centroid MNI coordinates
Synesthetes > Controls					
Left VOTC	0.0034	*d* = 1.135	3.75	1808	−33, −51, −18
Age (negative correlation)					
Left lateral prefrontal	0.0024	*r* = 0.637	4.39	1910	−33, 52, 0
Left mesial prefrontal	0.017	*r* = 0.477	3.79	500	−4, 37, 13

VOTC: ventral occipitotemporal cortex.

Significant clusters identified with a general linear model including group, age, gender, group × age and group × gender, using t-contrasts (df = 28). Cluster-forming threshold *P* < 0.001 (uncorrected); cluster-level significance corrected for multiple comparisons across the whole cortical surface using random field theory (RFT, corrected *P* < 0.05). For each cluster the table reports the anatomical location, the RFT-corrected cluster-wise *P* value, Cohen’s *d* effect size, the peak *Z* value (normal deviate derived from *t*(28)), the cluster size in vertices and the centroid MNI coordinates. *N* = 17 per group.

No significant group difference was found in cortical thickness or curvature.

The fusiform gyrus is subdivided into four architectonically distinct territories (FG1–FG4; Fig. 2B) based on cell density, laminar structure and other indices.^20,21^ The VWFA mostly overlaps with FG4.^22^ To study the topography of the increase in cortical area in this framework, we carried out a repeated-measures ANOVA, with the four subdivisions of the fusiform gyrus as the within-subject factor, plus the previous factors of group, age, gender, group × gender and group × age. There was no significant group × region interaction (*F*(3,84) = 0.81, *P* = 0.45), indicating that the increase in cortical area in synesthetes did not differ across fusiform subdivisions (Fig. 2C). We note that per-ROI group comparisons would be circular, as the ROIs were selected based on their overlap with the significant group-level cluster; we therefore report only the interaction, which is independent of the selection criterion.

We carried out a similar ROI analysis in the pSTG/SMG, the other candidate regions thought to be involved in TTS, a region in which whole-brain analysis showed no group difference. This null effect was found again in the ROI analysis (Student’s *t*-test: *t* = 1.49, *P* = 0.15). An additional Jeffreys–Zellner–Siow (JZS) Bayesian *t*-test^23^ found a BF_10_ = 0.766, in the 0.33–1 interval, indicating weak evidence for no group difference.^24^

Finally, we conducted an exploratory analysis of individual variability (see Supplementary Result S2 and Supplementary Figs S2–S4 for a full description). For each synesthete, we located the individual peak of excess in cortical surface area within the group-level fusiform cluster. This excess was larger in individuals with a more posterior peak along the fusiform axis. Future research may elucidate whether this variability is associated with subtle phenotypic differences among synesthetes.

### Effect of age

There was a main effect of age such that, across both groups, cortical surface area decreased significantly with age in mesial and lateral left prefrontal clusters (Fig. 3 and Table 1). No significant age effect was found for thickness or curvature. No significant group × age interaction was found for any measure.

**Figure 3:**
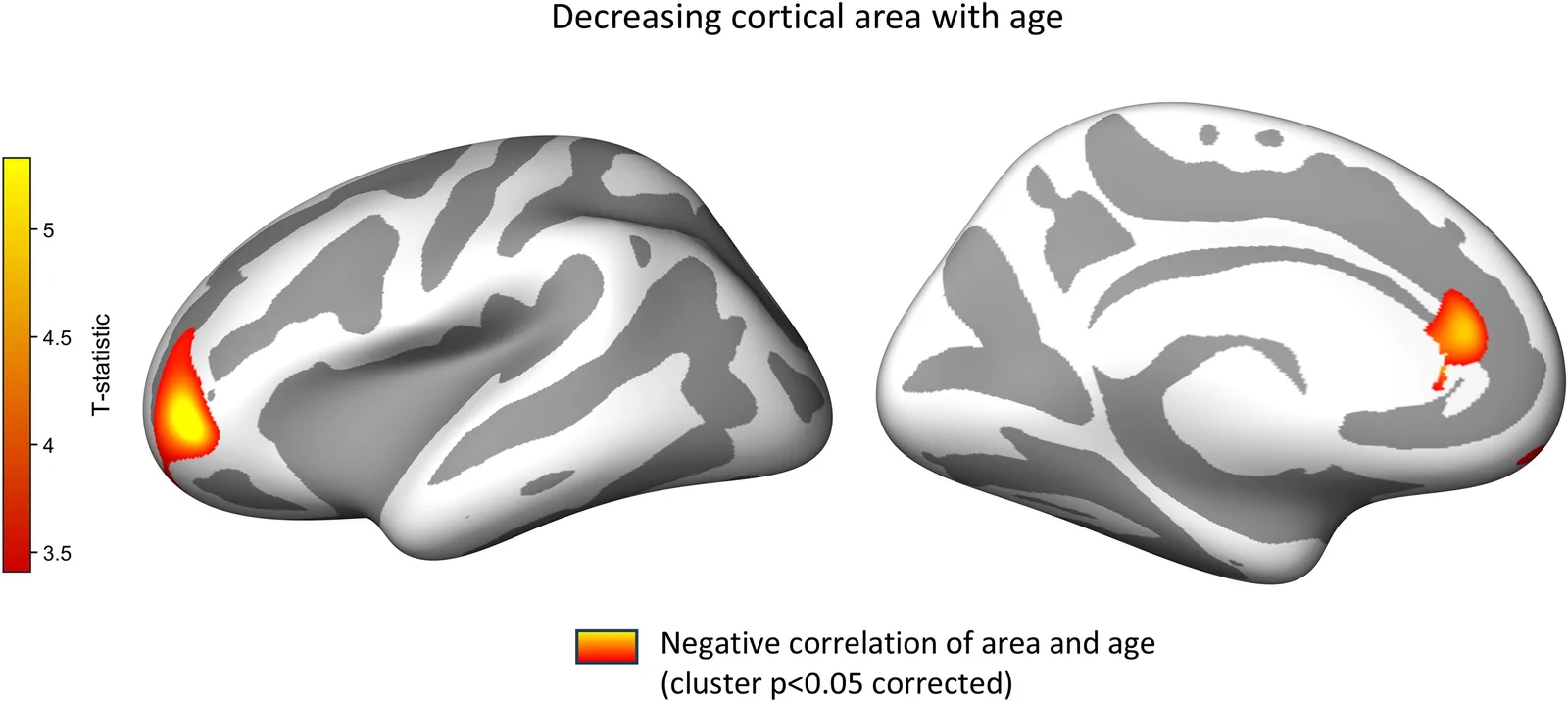
**Effects of age on cortical surface area in all participants (*N* = 34).** Statistical analysis: vertex-wise general linear model including group, age, gender, group × age and group × gender, with a t-contrast testing the effect of age across all participants (df = 28). Cluster-level inference used random field theory (RFT). Negative correlation of cortical surface area with age in the left prefrontal cortex (hot; cluster-forming threshold *P* < 0.001, cluster-level threshold RFT-corrected *P* < 0.05; lateral prefrontal peak *Z* = 4.63, cluster-level corrected *P* = 0.0024; mesial prefrontal peak *Z* = 3.94, cluster-level corrected *P* = 0.017). The colour bar indicates *t* values.

### Gender effect

Due to the very small number of male participants (*n* = 3 per group), effects of gender are unreliable and are reported in Supplementary Result S3, Supplementary Fig. S5 and Supplementary Table S2.

## Discussion

### Summary

The main finding of this study is that ticker-tape synesthetes show increased cortical surface area in the left fusiform region, compared with matched controls. This difference was specific to cortical surface area, as no group difference was found for cortical thickness or curvature.

We acknowledge that our sample size is relatively small, and that this study is therefore sensitive only to large effects. The great rarity of ticker-tape synesthesia and the lack of previous studies make an a priori power analysis impossible and strictly limit the number of available participants regardless of resources. In this context, our sample is comparable to or larger than those reported in morphometric studies of synesthesia.^12-15^ Future replication in larger samples will be needed to confirm the present findings and to detect potentially smaller effects in regions such as the pSTG/SMG where we report a null result.

Moreover surface area decreased with age in two left prefrontal clusters, consistent with the known pattern of cortical atrophy with ageing, which predominates in prefrontal regions.^25^ This effect did not interact with group, suggesting a similar trajectory of cortical ageing in synesthetes and controls.

The absence of a significant age effect for cortical thickness is somewhat unexpected given the overall age-related thickness decrease.^25,26^ This likely reflects limited statistical power given the relatively young and homogeneous age distribution of our sample, as age-related thickness changes are more prominent in younger and older cohorts.^26^

Our results appear to contrast with Ward *et al*.,^11^ who reported widespread structural differences across the whole brain between synesthetes and controls. However the two studies differ in important points of methods, which limits the validity of a direct comparison. First, Ward *et al*.^11^ pooled all types of synesthetes, including about one third of TTS. Second, their sample size was much larger, with 102 synesthetes. Third, they used 13 morphometric measures, surface area and thickness being the only ones shared with the present study. Among other findings, they report an increase in surface area in various temporal regions. Whether the fusiform area increase we observe in TTS generalizes to other synesthetic populations remains an open question.

Rather than pooling participants with different types of synesthesia, which may involve different neural mechanisms and behavioural profiles, we favoured a strategy of characterizing the pathophysiology of one well-defined type in depth, with the expectation that mechanistic commonalities will emerge from subsequent comparisons across types. TTS offers a particularly tractable case because its neural substrate overlaps with the well-characterized reading network, making it possible to formulate and test precise mechanistic hypotheses.

We discuss the anatomical location of the fusiform increase in area, in relation to the VWFA, its convergence with previous functional and connectional findings in TTS, the developmental implications of an area-specific effect and the parallel with other neurodevelopmental variants affecting the reading system.

### Anatomical location: the fusiform gyrus and the VWFA

The significant cluster was centred on the left mid-fusiform sulcus, overlapping with cytoarchitectonic areas FG1–FG4.^20,21^ This location is just medial to the VWFA, as functionally defined both by its increased activation in synesthetes than in controls during speech perception, and by its activation by printed words relative to other images.^5^ At lower statistical thresholds, the increase in area extended laterally to encompass the VWFA territory itself, within the OTS.

Moreover, the cluster also covered a more mesial region involved in reading, which we have previously proposed to label the Grapheme-Related Area (GRA).^27^ The GRA, which is located precisely within the third, anteromesial, subdivision of the fusiform gyrus FG3 (Fig. 2B; MNI −32 −58 −14),^28^ is defined by its role in encoding graphemes, i.e. single or multiple letters representing one phoneme (e.g. the English graphemes EE, SH and OUGH represent phonemes/i:/,/ʃ/ and/uː/). Moreover, contrary to the VWFA proper, the GRA is not sensitive to the distinction between words and pseudowords, while it is more sensitive to word length,^27^ supporting its involvement in phonological reading based on letters-sounds correspondence. A prerequisite for TTS to occur is the perception of phonologically permissible sequences, regardless of whether they are real words or not.^3^ This phonological rather than lexical dependency implicates the grapheme-phoneme conversion route, with the GRA as its endpoint in the VOTC.

It is worth noting that no morphological difference was found in the pSTG/SMG, the other major node of the TTS network. This could reflect lower statistical power to detect a subtle effect in this region, or it could indicate that the morphological predisposition to TTS is specific to the VOTC, while the dorsal phonological regions contribute to TTS through their input to the VOTC rather than their structural features.

The left lateralization of our finding follows from the functional anatomy of reading. The VWFA is strongly left-lateralized. Hence it is individuals with a structurally expanded left, but not right, fusiform cortex who are predisposed to develop TTS. As to the deeper causes of the left lateralization of the VWFA, these are generally attributed to the pull of left-lateralized language areas, which drive the functional specialization of the left VOTC during reading acquisition.^29,30^

### TTS and nonveridical perception

#### TTS and hallucinations

The role of the occipitotemporal cortex in the generation of nonveridical perception beyond TTS finds support in studies of hallucinations, although the diversity of mechanisms involved precludes simple generalizations.^31^ First, in Charles Bonnet syndrome, Ffytche *et al*.^32^ showed that hallucinations were accompanied by activation of visual areas whose specialization matched the content of the experience, suggesting that VOTC hyperexcitability can generate vivid visual percepts in the absence of external input. Second, in Parkinson’s disease, Dujardin *et al*.^33^ reported increased activation and connectivity in visual networks during hallucinations, encompassing the fusiform gyri bilaterally. In a meta-analysis, Vignando *et al*.^34^ found reduced cortical area in the occipitotemporal cortex in patients with hallucinations, suggesting that structural properties of this region modulate hallucinatory propensity. Third, in psychosis, van Ommen *et al*.^35^ observed activation of higher-order visual areas during hallucinations, and Rollins *et al*.^36^ found grey matter increases in the bilateral fusiform gyrus in patients with hallucinations. The possible link with TTS is further illustrated by our report of a patient in whom a psychotic episode transiently unmasked an additional ticker-tape synesthesia,^37^ suggesting that the substrate predisposing to TTS can be rendered symptomatic by pathological increases in cortical excitability.

#### TTS and other synesthetic features

The cluster of increased cortical area is centred on the mid-fusiform sulcus, an essential landmark with architectonic and functional significance and key integrative functions.^38^ It is surrounded not only by regions involved in reading (the VWFA and the GRA) but also territories associated with the processing of faces,^39^ bodies,^40^ objects^41^ or colour,^42,43^ among others. The broad, potentially domain-general, increase in surface area which we observed may be relevant to two observations. First, about two-thirds of TTS synesthetes also report other types of synesthesia, many with a visual component such as grapheme-colour or sound-colour associations.^3^ The anatomical differences observed here may indeed relate to ticker-tape synesthesia specifically, or to a broader predisposition towards multiple synesthetic experiences related to the ventral visual stream. Second, synesthetes in general tend to display enhanced visual mental imagery.^44-46^ An enlarged integrative fusiform territory could provide a richer visual representational substrate supporting the vivid imagery that characterizes synesthesia, but also the quality of usual mental imagery.

### Convergence with previous findings in TTS

The present finding adds cortical morphology to a converging set of structural and functional atypical features in TTS, mostly centred on the reading network and specifically on the left VOTC. In a series of studies, we previously showed that during speech perception, ticker-tape synesthetes over-activate a set of left-predominant regions overlapping with the reading network, including the VWFA and the left pSTG/SMG, two regions that show increased functional connectivity during speech perception.^5^ Anatomically, synesthetes show increased fibre density in the posterior segment of the arcuate fasciculus, specifically in the white matter underlying the pSTG/SMG, and the tractogram of this region reaches the VWFA ventrally.^6^ The present finding of increased cortical area in the fusiform region thus completes the picture at the structural level. The left VOTC is the one node of the TTS network for which all four types of evidence now converge: functional activation, functional connectivity, anatomical connectivity and now cortical morphology. Still, we note that the empirical findings on TTS come largely from our own group, reflecting the scarcity of independent work on TTS to date; replication by other teams is thus clearly needed.

### Structural predispositions: from connectivity to cortical area

When looking for the primary causal factor of TTS, it is tempting to implicate the deviant patterns in cortical morphology or structural connectivity. We previously argued that increased connectivity through the posterior arcuate fasciculus could constitute such a causal factor.^6^ However, this argument is far from conclusive, as it is well established that practice and experience can induce changes in white matter microstructure (for reviews see^47,48^). This is the case also in the domain of reading. Thus literacy strengthens the connectivity of the VWFA through the arcuate fasciculus.^49^ Although ticker-tape synesthetes do not differ from controls in their practice of reading, the synesthetic experience itself, with decades of involuntary visualization of orthographic forms, could conceivably have reinforced the anatomical connections that support this visualization, making it difficult to determine whether increased connectivity is a cause or a consequence of TTS. The present finding of increased cortical surface area in the left fusiform region offers a more compelling candidate for a primary structural predisposition, for three converging reasons.

First, an increase in cortical surface area rather than in cortical thickness carries strong implications regarding its developmental origin. Area and thickness are uncorrelated and genetically independent traits,^17^ and genome-wide association studies showed that genetic influences are more numerous and more powerful for area than for thickness.^16^ Importantly, the genes associated with area are preferentially expressed during prenatal development in neural progenitor cells.^16^ According to the radial unit hypothesis,^50,51^ area is determined by the number of cortical columns generated through divisions of progenitor cells during early embryogenesis. Cortical thickness, by contrast, reflects postnatal processes susceptible to experience-dependent plasticity.^52,53^ Thus increased thickness has been documented in the motor cortex following juggling training^54^ or in auditory regions of musicians.^55^ The increase in fusiform surface area that we observe in TTS synesthetes is therefore unlikely to result from any form of practice or expertise.

Second, *reduced* cortical surface area was found in the very same region in adults with dyslexia, a condition that involves neurodevelopmental deviations with a prenatal and genetic origin.^56^ In children with developmental language disorder, including dyslexia, Bahar *et al*.^57^ found smaller surface area in the fusiform gyrus, with a centroid (MNI −33, −56, −16), located 5 mm away from the centroid of the cluster we found. Cortical thickness did not differ between groups. Such surface area anomalies in the fusiform cortex have been detected in pre-reading children at familial risk for dyslexia, that is before any reading instruction.^58^ One may speculate that while impoverished reading is associated with reduced area in the left fusiform gyrus, an enhanced and atypical function such as in TTS is associated with increased area in the same territory.

A third, less conclusive, argument is that the cortical territory showing increased area in synesthetes extends far beyond the functionally defined reading network. The significant cluster encompasses most of the fusiform gyrus, a territory that subserves not only reading but also the processing of many other visual features and categories, as discussed before. If the morphological difference were a consequence of TTS rather than its cause, we would expect it to be restricted to the reading-specific part of the occipito-temporal cortex.

Taken together, these three lines of evidence suggest that the increased fusiform area in TTS reflects an innate predisposition of the cortical territory on which orthographic representations later develop, rather than a consequence of the synesthetic experience itself. We do not dismiss the dominant connectivity-based account of synesthesia,^9,11,59^ and we have indeed documented increased functional and anatomical connectivity in TTS.^5,6^ What we raise as a possibility is that at least part of the atypical connectivity may be a downstream consequence of the cortical expansion, rather than an independent primary cause. Finally, our observation is limited to TTS, and atypical connectivity may well play a more decisive primary role in other forms of synesthesia.

### Top-down influence on the VOTC

We propose that during reading acquisition, the enlarged fusiform territory of synesthetes develops more detailed or more easily activable orthographic representations. After literacy is established, top-down phonological signals flowing through the enhanced arcuate fasciculus pathway automatically activate the enriched orthographic representations, giving rise to the ticker-tape experience. A small proportion of synesthetes report perceiving letters as if projected in the external space,^3^ and evidence from grapheme-colour synesthesia suggest that the interplay of top-down and bottom-up influences may differ between so-called projectors and associators.^10^ The anatomical correlates of this distinction may deserve further study.

An ensuing prediction is that, using time-resolved techniques such as MEG/EEG, the first divergence between synesthetes and controls during speech perception should occur in the left VOTC, as soon as it is activated by auditory input, which may occur from 100 to 150 ms after speech onset.^60^

A recent study combining artificial neural network modelling and human brain lesion data showed that a model trained with sentence-level language alignment explained human VOTC activity better than a purely visual model, and that this advantage was left-lateralized.^61^ In stroke patients with damage to white matter tracts connecting the VOTC to language areas, the language-trained model’s advantage disappeared while the purely visual model’s fit increased. Those findings support our hypothesis that the strength of language influence on the VOTC, diminished after stroke, is enhanced in ticker-tape synesthesia, and that cortical area in the left fusiform region is one of the determinants of this language-vision interaction.

### The advantage of being a synesthete

One might expect that an enlarged fusiform cortex would confer measurable orthographic advantages. Indeed, in a behavioural study,^62^ we compared 22 TTS synesthetes to 22 matched controls on tasks relying on orthographic working memory. Synesthetes were significantly faster and more accurate than controls in letter counting, backward spelling and letter shape decision on auditory input, all tasks in which the vivid and stable orthographic imagery of TTS provides an internal written support. However, synesthetes did *not* differ from controls in tasks with a visual written input, indicating that the enlarged fusiform cortex is not simply more excitable for all stimuli, but becomes preferentially engaged through the phonological-to-orthographic pathway that drives the synesthetic experience.

## Conclusion

Ticker-tape synesthetes show increased cortical surface area in the left fusiform gyrus, encompassing the VWFA and the graphemic representation area. This finding converges with previously documented increases in functional activation, functional connectivity and anatomical connectivity to establish the left VOTC as the node where all structural and functional signatures of TTS coincide. Three independent arguments (the genetic and prenatal determination of cortical surface area, the mirror-image pattern observed in developmental dyslexia and the spatial extent of the area increase beyond the reading network) suggest that this morphological difference constitutes an innate predisposition rather than a consequence of the synesthetic experience. We propose that a genetically determined expansion of fusiform cortex provides a richer substrate for orthographic representations, which are then excessively activated by top-down phonological input, giving rise to the vivid and involuntary visualization of speech.

## Supplementary Material

fcag408_Supplementary_Data

## Data Availability

The anonymized individual FreeSurfer maps and the analysis code are available on https://zenodo.org/records/22172039.
