# Supplementary material for: Increased left fusiform surface area: a structural predisposition to ticker-tape synesthesia
Source: Brain Commun. 2026 Oct 27;8(6):fcag408. doi: 10.1093/braincomms/fcag408 (PMC13748745; doi:10.1093/braincomms/fcag408)
Supplement: Supplementary file 1 [file fcag408_supplementary_data.docx]

# Supplementary material

## Supplementary result 1: Robustness for smoothing parameters: p. 2

## Supplementary result 2: Individual variability of area excess in synesthetes: p.4

## Supplementary result 3: Effects of gender on cortical area: p. 7

# Supplementary result 1: Robustness for smoothing parameters

## Smoothing: 15 mm field width at half maximum (fwhm)

The comparison of cortical surface area between synesthetes and controls carried out after smoothing the data with a kernel of 15 mm fwhm gave results essentially identical to those reported in the main text after smoothing at 20 mm fwhm, including the core finding of increased surface area in the left fusiform region (Supplementary Table 1 and Supplementary Figure 1).

### Supplementary Table 1

|  | Cluster  corrected p value | Peak voxel  Z value | Cluster size  # vertices | Centroid  MNI coordinates |
| --- | --- | --- | --- | --- |
| Synesthetes > Controls |  |  |  |  |
| Left VOTC | 0.0058 | 3.88 | 989 | -29,-63, -9 |
| Age (negative correlation) |  |  |  |  |
| Left lateral prefrontal | 0.0028 | 4.29 | 1233 | -40, 54, -2 |
| Left mesial prefrontal | 0.030 | 4.21 | 459 | -5, 41, 15 |

*Whole-brain analysis (smoothing with a kernel of 15 mm fwhm). Significant differences in cortical area (cluster-forming threshold p < 0.001; corrected cluster p < 0.05). N = 17 per group. VOTC: ventral occipitotemporal cortex.*

### Supplementary Figure 1

***Effect of smoothing kernel.*** *When smoothing at 15 mm FWHM, the same fusiform region of increased surface area was found in synesthetes (hot; vertex-wise general linear model, two-sample t-contrast comparing synesthetes and controls, df = 28; cluster-forming threshold p < 0.001, cluster-level threshold RFT-corrected p < 0.05; peak t(28) = 4.17, cluster-level corrected p = 0.006; N = 17 per group), although somewhat smaller than when smoothing at 20 mm FWHM. The color bar indicates t values. RFT: random field theory.*

## Smoothing: 10 mm fwhm

The same analyses carried out after smoothing at 10 mm fwhm produced qualitatively similar results. The cortical patch of increased surface area in the fusiform region was split into 3 smaller clusters, which did not reach cluster-level significance after RFT correction.

# Supplementary result 2: Individual variability of area excess in synesthetes

An exploratory analysis of individual variability in peak location was conducted. For each synesthete, we computed a difference map (individual area minus mean control area) within the significant fusiform cluster, smoothed it within the cluster using 5 iterations of neighbor averaging, and identified the peak of maximal excess. A principal components analysis (PCA) on the MNI coordinates of these individual peaks revealed that 77% of the spatial variance was captured by a single axis oriented predominantly in the anterior-posterior direction (PC1 loadings: x = -0.26, y = +0.89, z = -0.36), consistent with the long axis of the fusiform gyrus (see Supplementary Figure 2). Individual peaks were distributed along this axis with a spread of 13.3 mm (SD of PC1 scores), reflecting substantial inter-individual variability in the precise locus of maximal area excess within the fusiform region.

### Supplementary Figure 2

***Individual variability of area excess in synesthetes.*** *Individual peaks were distributed along an anteroposterior axis with substantial inter-individual variability in the precise locus of maximal area excess within the fusiform region. PCA: principal components analysis. N = 17 per group.*

Interestingly, position along this axis correlated with the magnitude of area excess (r = -0.51, p = .038), with more posterior peaks associated with larger differences from controls (see Supplementary Figures 3 and 4). Future research may elucidate whether such variability is associated with subtle phenotypic variability among synesthetes.

### Supplementary Figure 3

***Individual variability of area excess in synesthetes.*** *Synesthetes with more posterior peaks of area increase also had a larger area increase relative to controls. . N = 17 per group. r : Pearson’s correlation coefficient.*

### Supplementary Figure 4

**Individual area excess in synesthetes within the fusiform cluster**

***Individual variability of area excess in synesthetes.*** *Map of area excess (smoothed per-vertex surface area in mm²) in individual synesthetes relative to the mean of controls within the fusiform cluster of increased surface area. Blue: Synesthetes below controls; red: synesthetes above controls. Individual peaks are labeled with a black cross.*

# Supplementary result 3: Effects of gender on cortical area

Gender and the group × gender interaction were included in the GLM described in the main text because failing to model known sources of variance inflates the residual error term and reduces sensitivity for the contrasts of primary interest. However the small number of male participants (n = 3 per group) means that any result involving gender, whether significant or not, cannot be safely interpreted (see Supplementary Figure 5 and Supplementary Table 2).

### Supplementary Figure 5

***Effects of gender on cortical surface area.*** *Statistical analysis: vertex-wise general linear model including group, age, gender, group × age and group × gender (df = 28); cluster-level inference using random field theory (RFT). Larger cortical surface area was found in males than in females in bilateral mesial and lateral prefrontal areas (hot; main effect of gender, t-contrast males > females; cluster-forming threshold p < 0.001, cluster-level threshold RFT-corrected p < 0.05). This difference was larger in controls than in synesthetes in a left orbitofrontal region (blue; group × gender interaction t-contrast). N = 17 (including 3 males) per group. The color bar indicates t values.*

### Supplementary Table 2

| Effects of gender on cortical area | | | |
| --- | --- | --- | --- |
|  | Peak t value | Peak MNI coord. | p_RFT |
| Left hemisphere | | | |
| Male > Female | | | |
|  | 4.286 | -2, -44, 25 | < 0.001 |
|  | 6.806 | -2, 44, -26 | < 0.001 |
|  | 5.702 | -43, 51, -6 | 0.011 |
|  | | | |
| Male > Female in Controls > Synesthetes | | | |
|  | 5.092 | -2, 45, -26 | 0.027 |
|  | | | |
| Right hemisphere | | | |
| Male > Female | | | |
|  | 5.872 | 9, 25, 34 | < 0.001 |
|  | 4.134 | 48, -25, 62 | 0.033 |
|  | 3.926 | 25, 31, 36 | 0.020 |
|  | 4.965 | 49, 41,-11 | 0.015 |
|  | 4.508 | 24, -4, 50 | 0.025 |

*Effects of gender on cortical surface area. The table provides the peak t value, the peak MNI coordinates, and the clusterwise RFT-corrected p value of the clusters depicted in Supplementary Figure 5.*
